# Plato's Cave Algorithm: Inferring Functional Signaling Networks from Early Gene Expression Shadows

**DOI:** 10.1371/journal.pcbi.1000828

**Published:** 2010-06-24

**Authors:** Yishai Shimoni, Marc Y. Fink, Soon-gang Choi, Stuart C. Sealfon

**Affiliations:** Department of Neurology and Center for Translational Systems Biology, Mount Sinai School of Medicine, New York, New York, United States of America; University of Zurich, Switzerland

## Abstract

Improving the ability to reverse engineer biochemical networks is a major goal of systems biology. Lesions in signaling networks lead to alterations in gene expression, which in principle should allow network reconstruction. However, the information about the activity levels of signaling proteins conveyed in overall gene expression is limited by the complexity of gene expression dynamics and of regulatory network topology. Two observations provide the basis for overcoming this limitation: a. genes induced without de-novo protein synthesis (early genes) show a linear accumulation of product in the first hour after the change in the cell's state; b. The signaling components in the network largely function in the linear range of their stimulus-response curves. Therefore, unlike most genes or most time points, expression profiles of early genes at an early time point provide direct biochemical assays that represent the activity levels of upstream signaling components. Such expression data provide the basis for an efficient algorithm (Plato's Cave algorithm; PLACA) to reverse engineer functional signaling networks. Unlike conventional reverse engineering algorithms that use steady state values, PLACA uses stimulated early gene expression measurements associated with systematic perturbations of signaling components, without measuring the signaling components themselves. Besides the reverse engineered network, PLACA also identifies the genes detecting the functional interaction, thereby facilitating validation of the predicted functional network. Using simulated datasets, the algorithm is shown to be robust to experimental noise. Using experimental data obtained from gonadotropes, PLACA reverse engineered the interaction network of six perturbed signaling components. The network recapitulated many known interactions and identified novel functional interactions that were validated by further experiment. PLACA uses the results of experiments that are feasible for any signaling network to predict the functional topology of the network and to identify novel relationships.

## Author Summary

Elucidating the biochemical interactions in living cells is essential to understanding their behavior under various external conditions. Some of these interactions occur between signaling components with many active states, and their activity levels may be difficult to measure directly. However, most methods to reverse engineer interaction networks rely on measuring gene activity at steady state under various cellular stimuli. Such gene measurements therefore ignore the intermediate effects of signaling components, and cannot reliably convey the interactions between the signaling components themselves. We propose using the changes in activity of early genes shortly after the stimulus to infer the functional interactions between the unmeasured signaling components. The change in expression in such genes at these times is directly and linearly affected by the signaling components, since there is insufficient time for other genes to be transcribed and interfere with the early genes' expression. We present an algorithm that uses such measurements to reverse engineer the functional interaction network between signaling components, and also provides a means for testing these predictions. The algorithm therefore uses feasible experiments to reconstruct functional networks. We applied the algorithm to experimental measurements and uncovered known interactions, as well as novel interactions that were then confirmed experimentally.

## Introduction

A major goal of systems biology is to elucidate the molecular networks that underlie cellular decision-making and predict emergent properties of the system. Knowledge of molecular networks provides novel insight into the mechanisms underlying both physiological and pathological cellular processes. Such networks were constructed in yeast [1], [2], *Escherichia coli* [3]–[5], *Saccharomyces cerevisiae* [6] and human [7], [8], mostly using large-scale genetic manipulation in order to identify gene to gene interactions, non-coding RNA interactions, and gene to phenotype interactions. These networks were analyzed, and the function of several network components was elucidated [3], [4], [9]–[13].

High-throughput gene expression assays, such as microarrays and quantitative real-time PCR, provide insights into mechanisms mediating normal physiology and disease states. Gene assays have been used to identify novel genes associated with specific cellular events or phenotypes, and to unravel interaction networks between the genes. Still, for some of the important questions facing cell biologists, the statistical and mathematical approaches used to analyze these data are not applicable. Specifically, the activity state of many signaling components mediating the cellular response (e.g. some scaffold proteins or transcription factors) cannot be measured in systematic high throughput assays, and therefore the interactions between them are not directly decipherable by these approaches.

Several methods have been developed to reconstruct signaling networks from experimental data [14]. However, most of these methods rely on measuring the activity levels of the signaling components in question under several conditions, and therefore require a large number of experiments for each signaling component. This applies for bottom-up approaches that use experimental determination of individual biochemical interactions to reconstruct the network [15], as well as for many top-down approaches such as partial least squares (PLS) [16]–[18], modular response analysis (MRA) [19]–[23], many methods using Bayesian inference [24]–[28], and methods based on dynamic properties [29]–[31]. The few methods that do not require measuring the activity of the signaling components rely on creating large interaction databases by performing many experiments [32], or integration of large databases from several sources [33], [34]. Although the latter methods can be useful for finalizing well-studied networks, they are not appropriate when little data is available about the network.

Gene profiling has been previously used to find interactions between various molecules [32], [35], but the focus has been on late time points, when gene activity reaches a quasi-steady state. At these time-points the initial signal from the signaling component is partly degraded due to feedback and cross-talk between the genes. At later time points, the changes in expression of many genes may fail to show a simple function that correlates with the activity of the upstream signaling components responsible for regulating that gene [36]. Thus the use of gene expression measured more than ∼one hour after modulation of the system can provide a non-mechanistic pattern-matching representation of cellular state. However such approaches may not allow a quantitative reconstruction of the biochemical network in a way that is analogous to construction of a network using measurement of the protein activity states themselves.

**Figure 1:**
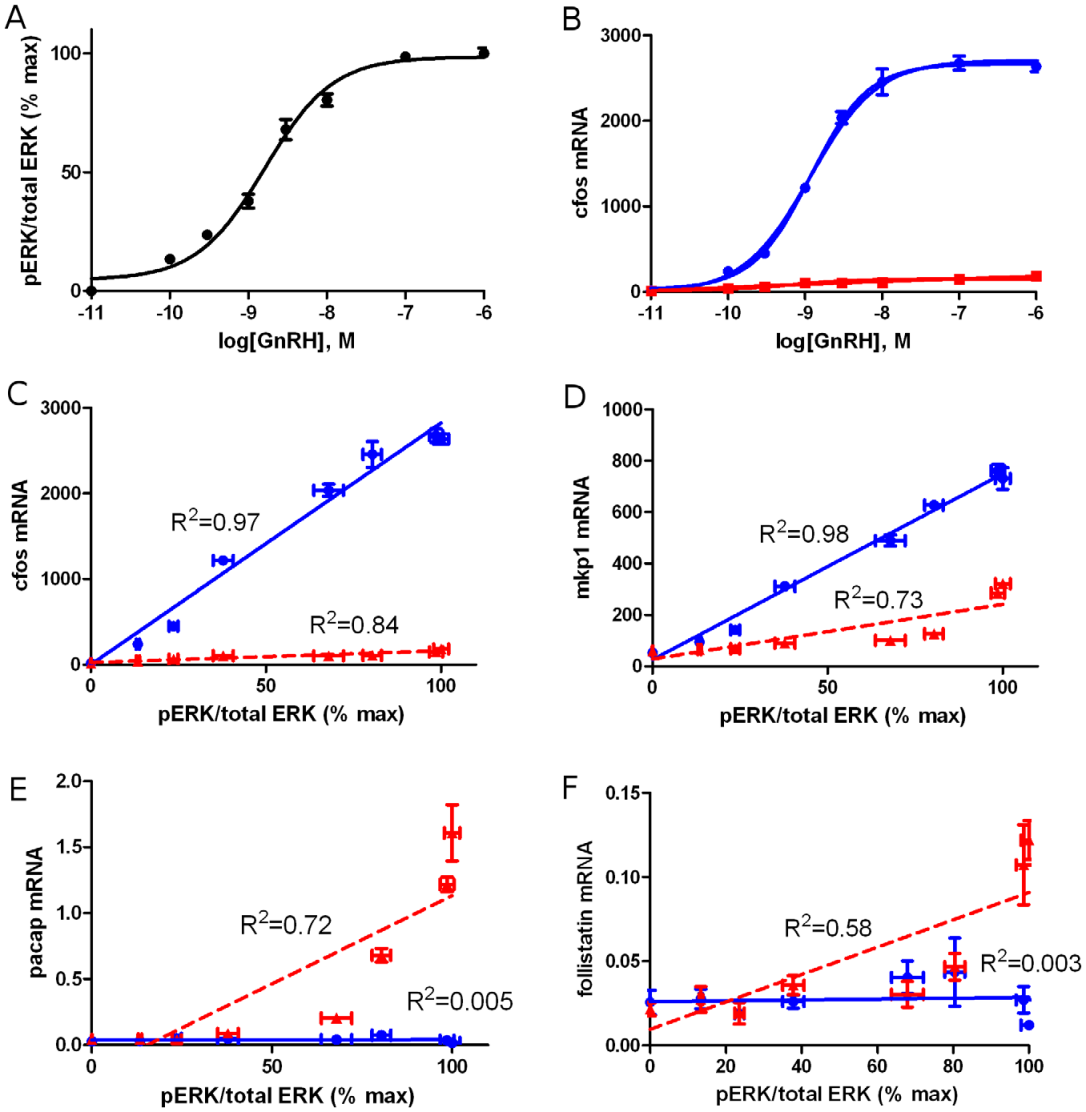
Response of early and late genes and their correlation to signaling activity. A. The normalized pERK level (Y axis) as a function of GnRH concentration (X axis) as determined by ERK ELISA assays at 5 minutes following activation of LβT2 gonadotropes. B. The expression level of cfos mRNA (Y axis) as a function of GnRH concentrations (X axis) as determined by quantitative PCR at 45 minutes (solid line) and 5 hours (dashed line) following GnRH stimulation of LβT2 gonadotropes. C–F. The expression level of several genes is plotted vs. the activity levels of pERK at 45 minutes (squares) and 5hrs (triangles) following activation of LβT2 gonadotropes by a number of different concentrations of GnRH. Both time-points are fitted to a linear curve (solid and dashed lines, respectively). C and D. The early genes such as cfos and mkp1 exhibit a good linear correlation to pERK activity at 45 minutes, and much weaker correlation at 5 hours. E and F. Later genes such as pacap and follistatin exhibit no correlation to pERK activity at 45 minutes and show weak correlation at 5 hours. Error bars are standard error of mean. n = 4 per point.

A potential technique to measure indirectly the activity levels of signaling components is presented by measuring the activity of early genes, which are defined as genes that do not require any *de*-novo synthesis in order to start their transcription. Specifically, their regulatory transcription factors are pre-formed and the activation states of these factors are altered by modulations in cellular signaling. As a result, their promoters act as direct, quantitative sensors of the cellular signaling state [36]–[39]. Such genes are thus the first genes to be induced following a change in the cell's condition, and are usually activated within minutes. To illustrate the linear function correlating signaling components and early genes measured at early time points, we exposed gonadotrope cells to the hormone GnRH at varying concentration and measured, the resulting levels of one active signaling molecule, phosphoERK, as well as the levels of transcripts for several early genes and non-early genes at 0.75 and 5 hours (Fig. 1). The results show that all of the early genes are linearly correlated with the levels of phosphoERK and the correlation is much higher, (R^2^ ranging from 0.92 to 0.99) when measured at 0.75 hours than when measured at 5 hours. Therefore, if additional experiments were performed, such as ERK inhibition, to determine which of these genes are most dependent on activation of ERK, the measurement of such genes would provide an indirect, yet sensitive and accurate measurement of the levels of phosphoERK. The relationship is most linear and most direct for early genes measured at early time points. In contrast, in secondary and tertiary genes, which require newly synthesized transcription factors or enhanceosome components to regulate their activity, their activity levels normally do not have a simple function relating it to the activity levels of upstream signaling components at any time point. Notably, the linear amplification between signals and early genes is the basis of the widespread use of synthetic gene reporter constructs to provide quantitative measurements which accurately reflect changes in cell signaling (e.g. using the activity of a cAMP response element reporter to reflect changes in adenylate cyclase activity). Because of these considerations, the utilization of early gene profiling provides an experimentally and computationally tractable approach to reverse engineer the interaction network of signaling components.

Here we present a robust and efficient algorithm named PLACA that uses high throughput assays of early gene expression at early time points combines with perturbation of cellular components in order to uncover experimentally verifiable functional interactions between the components upstream of these early genes. Notably, in addition to the reverse engineered network, PLACA also identifies the specific genes that manifest the functional interaction. Thus PLACA facilitates experiments to validate the inferred interactions.

We tested the performance of PLACA by reconstructing a synthetic network, and found that when using several independent experiments it is robust to experimental noise. Additionally, we studied the early gene responses to signaling component perturbations in the pituitary gonadotrope and used PLACA to reverse engineer the network of this crucial component of the reproductive system. Many of the inferred functional interaction have been previously observed, and novel functional interaction predictions were then successfully tested experimentally.

A web-interface for PLACA is available at http://tsb.mssm.edu/primeportal/?q=placa_prog.

## Methods

### Algorithm Methodology Overview

As stated above, current methods for inferring signaling regulatory networks from gene activity data are not suitable for high throughput experiments in large networks. This represents a significant bottleneck in translating readily obtainable cellular readouts such as mRNA levels into detailed network interaction maps. Some of the nodes within the signaling network are comprised of elements such as transcription factors and scaffold proteins for which it is difficult to obtain activity measurements systematically. Even in the case of kinases, where many active state antibodies exist, other activity states may not be well characterized. Therefore we have developed a robust and efficient algorithm for the analysis of the interactions between signaling components, based on the activity level of early genes that are downstream of these signaling components. This algorithm infers the activity of the signaling components indirectly from measurement of the activity of early genes. By analogy to the allegory in which reality is perceived indirectly via shadows cast on the wall of the cavern we inhabit, we refer to this as “Plato's Cave Algorithm”, or PLACA.

**Figure 2:**
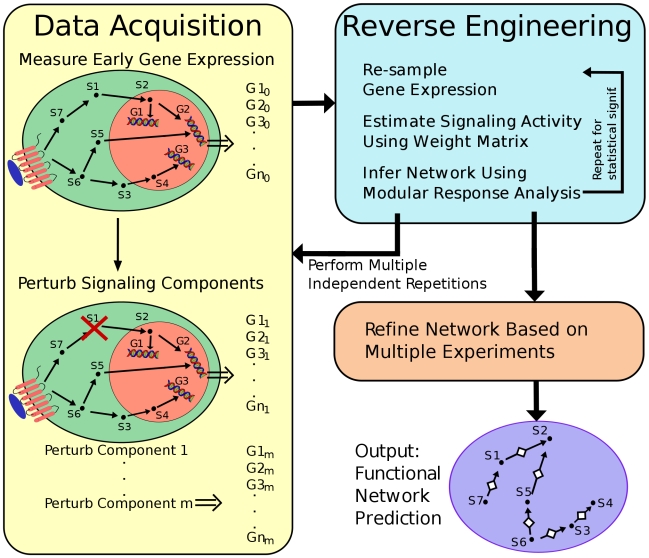
PLACA methodology overview. The flow chart describes the methodology proposed in Plato's Cave Algorithm. The algorithm is made of three stages: Data acquisition; Reverse Engineering that is repeated several times for statistical significance; and Post-processing where the results of several experiments are integrated.

PLACA is a multi-step algorithm based on an integration of well-established techniques. We outline the practical steps needed to use PLACA in order to uncover the functional interactions between signaling components, after choosing a list of *n* signaling components, and a set of *m* early genes that are predicted or known to be affected by the signaling components. Fig. 2 illustrates how these steps can be divided into 3 stages: experimental data acquisition, reverse engineering, and post processing. These stages are briefly explained here, and described in more detail in the following sub-sections.

The data acquisition stage for PLACA consists of *n*+1 experiments. The first experiment measures the mean activity and the standard deviation of the activity of all early genes. In the following *n* experiments each signaling component is perturbed in turn, and the activity and standard deviation of the activity of all the early genes are measured. Next, the reverse engineering stage is performed. First, a weight matrix describing the connections between genes and signaling components is calculated, and used to obtain an estimate of the change in activity of each signaling components following each perturbation. The estimated change in activity is used to infer the interactions between the signaling components by applying a reverse engineering method, and we chose to use MRA [19]–[23] for reasons that are detailed below. In order to achieve higher statistical significance of the results, the signaling activity estimation and MRA are repeated several times using a data re-sampling technique. Using the results obtained by re-sampling, only interactions with sufficient statistical significance are retained. Finally, the post-processing stage is applied if several independent experimental results of similar experiments are available, in which case interactions that do not appear in a sufficient number of the experiments are excluded. See Supplementary text S1 for technical details on the post processing stage.

### Data Acquisition

The algorithm is applied to the mean activity levels and the standard deviation in activity levels of early genes. These are performed both under normal conditions, and following perturbation of each signaling component. Experimentally, these perturbations can be performed using chemical inhibitors (e.g. kinase inhibitors, protease inhibitors, or channel blockers), siRNA, expression of over-active or dominant negative constructs, or over expression of the gene. The activity levels of the genes can be measured using quantitative real time PCR or microarrays. The activity level of a gene may be the fold-change of the individual transcript compared to some gene, its concentration, or its copy number/cell. We use the activity of early genes as an estimate for signaling component activity, and derive a weight matrix (as explained below) representing how much the change in activity of each early gene contributes to the estimated change in activity of each signaling component.

Specifically, if the change in gene activity after each perturbation is stored in matrix *ΔG*, and the weight matrix is denoted by *W*, then the estimated change in activity of the signaling components following each perturbation is given by *ΔS = W(ΔG)^T^*. This assumes a linear connection between signaling component activity and gene activity, as was observed experimentally. Mathematically, this is equivalent to assuming that the change in the activity of an early gene depends linearly on the weighted sum of the changes in the activity of the signaling components. The linearity assumption can also be justified by considering small changes from maximal (or quasi steady-state) values of the early gene activity.

### Weight Matrix Determination and Refinement

#### Weight matrix construction

To obtain the weight matrix, we face the challenge of identifying genes that change only following certain signaling component perturbations, where this change is not likely attributed to experimental noise or to internal noise in the gene expression. Such genes are ideal markers for the activity estimation of the signaling components that caused the change. To this end we use three scoring functions for each gene. First we define the *activity score*, which estimates the relative magnitude of the change in the activity level of each gene following each perturbation. Next we define a *P-score* that estimates the probability that the change in activity does not results from noise. Finally, we define the information score, which takes under consideration the changes in activity following every perturbation (the activity fingerprint of the gene), and gives the highest score to genes that carry information that is specific to a single perturbation. We assume that the change in activity level, the noise levels, and the specificity of each gene are independent quantities. Although the scores are not true probabilities, in order to reflect this assumption in the final score, we multiply the three scores to obtain the final weight matrix. We note that there is obviously some correlation between noisy genes and a large change in activity. However, by assuming independence we give a preference to non-noisy genes that manifest a large change in activity, thus making the overall score a bit more restrictive.

**Figure 3:**
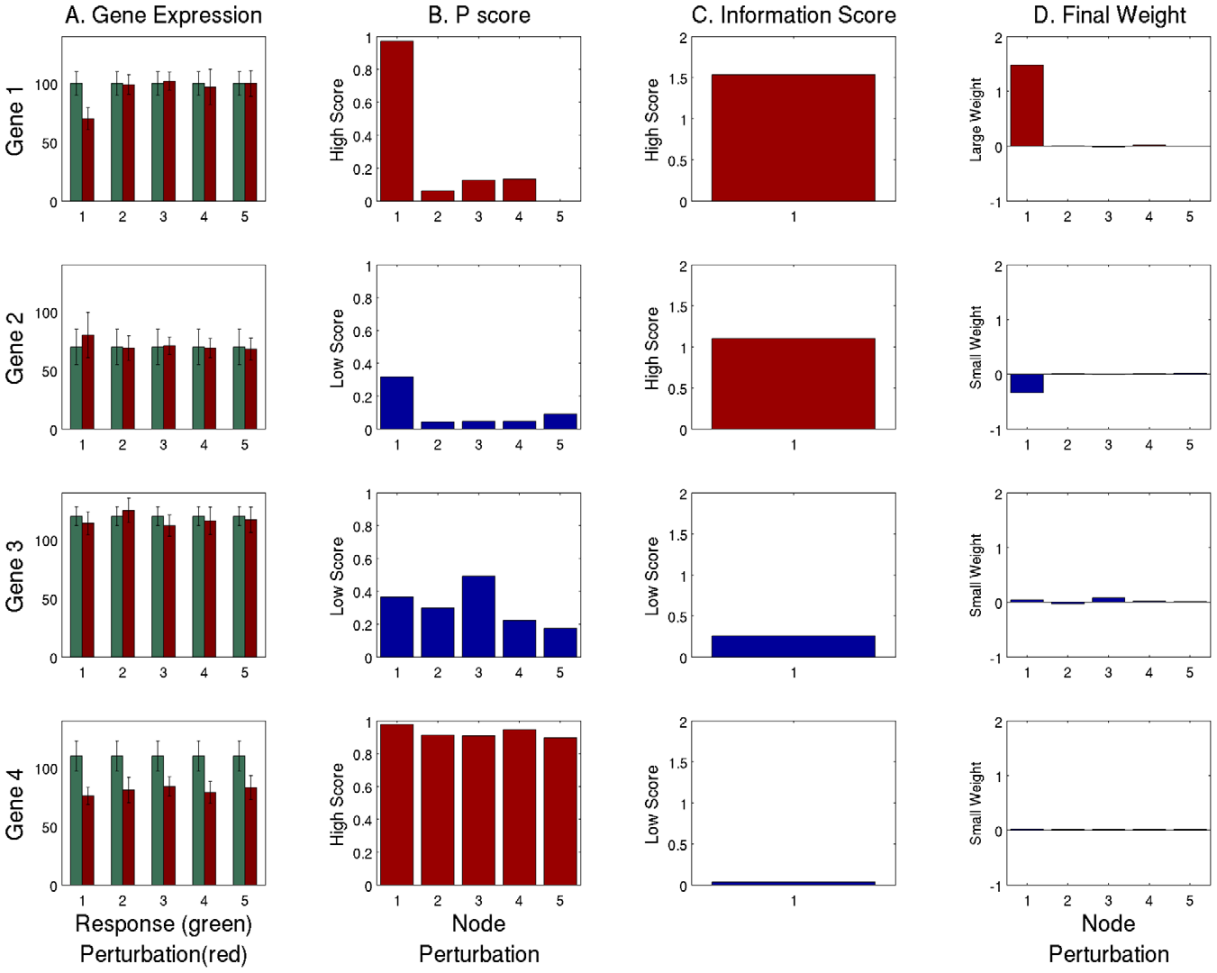
Weight Matrix Derivation – choosing good predictors of signaling activity. A. The expression levels (in arbitrary units) of four genes under five perturbations (red bars), and their levels without any perturbation (green bars); B. The Z-values for each gene under each perturbation. High Z-values are obtained for genes with statistically significant change (red bars), and low Z-values are obtained when the change can be attributed to noise (blue bars); C. The information score of each gene. When the change in gene activity is specific to one perturbation the information score is high (red bars), and otherwise it is low (blue bars); D. The final weight attributed to each gene for each perturbation. A gene can only get a high weight (red bars) if it has both a high Z-value and a high information score. The values used for the gene expression are not drawn from any experiment and were generated merely to illustrate the methodology.

Fig. 3 illustrates the determination of P-scores, information scores and the final weights derived from four hypothetical gene expression levels following perturbations of five signaling components. Genes showing low noise levels (high P scores) and higher specificity for particular perturbations (high information scores) are weighed more highly in the matrix that will be used for predicting the functional interactions of the network.

#### Activity score

The activity score for each gene is the change in activity for that gene following each perturbation, multiplied by the sign of the perturbation (inhibition or activation of the signaling component), and normalized across perturbations. This is done to avoid biasing the results to favor genes that have a very low initial activity (as might occur by normalizing according to initial activity, e.g. fold-change), or biasing them towards highly expressed genes (by considering the absolute change in gene activity). A high absolute activity score means a large change in the activity of the gene was caused by the specific perturbation when compared to other perturbations. Let us assume *n* signaling components and *m* early genes. We denote the mean activity of gene *k* (*k* = 1..*m*) under normal conditions by *A_k_*, and after perturbation of signaling component *j* (*j* = 1..*n*) by *G_jk_*. The change in activity is *C_jk_ = A_k_−G_jk_*. The normalized change in activity gives the activity score, given by
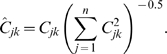



#### P-score

The P-score indicates the probability that the change in the mean expression is not due to noise. A P-score close to unity means that it is unlikely that the measured change in the mean activity is due to noise, and a P-score close to zero means that the change in activity can be attributed to measurement noise. We use a standard two-sample t-test to obtain the P-score. For completeness we introduce here the intermediate stages used in our calculations, and introduce nomenclature that is used in the following sections. To construct the P-score we use an intermediate Z-value, assuming that the distribution of the gene activity levels is close to Gaussian. In the case of gene expression levels, it was shown that many genes follow a log-normal distribution [40]. Therefore, to get a Gaussian distribution we use the mean of the logarithm of the activity following a perturbation, denoted by *LogG*. We denote the standard deviation of *LogG* (divided by the square root of the number of measurements to apply for a t-test) by *dG*. Similarly, we denote the mean of the logarithm of the normal activity of gene *k* by *LogA_k_*, and the corrected standard deviation of *LogA_k_* measurement by *dA_k_*. The Z-value of the change in gene *k* due to a perturbation in signaling component *j* is given by
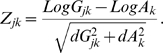



The P-score *P_jk_* is simply 1 minus the P-value of *Z_jk_*. Specifically, the P-score is given by twice the cumulative distribution function of N(0,1) (i.e. the normal distribution with a mean of zero and standard deviation of one), from zero to the absolute value of *Z_jk_*. The null hypothesis is that the samples giving *A_k_* and *G_jk_* are drawn from Gaussian distributions with different means. Therefore P-score approaching one indicates a high probability that the change in gene expression signifies a real change in the mean expression and is not due to noise.

#### Information score

Ideally, to quantify the change in activity of a signaling component, we are looking for genes whose expression change following perturbation to only that signaling component, and not to other components in the network. The information score quantifies how close a gene is to that ideal. It is inspired by the Shannon entropy function [41], which is a measure of the average information contents in a message. The Shannon entropy function is originally applied to a set of probabilities, and so we must normalize the expression results across perturbations so that they will be non-negative and their sum will equal unity. To this end we take the set of Z-scores for each gene, square them to make them non-negative, and divide them by their sum. The normalized Z-score for gene *k* takes the form 
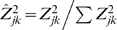
, where the sum is over *j* = 1..*n* . The information score function for gene *k* is then
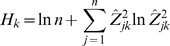
Due to the properties of the Shannon entropy function, a gene can get an information score of zero if (and only if) it changes equally significantly across all perturbations, and will get the highest score (of ln*n*) if it has a Z-value of zero for all the perturbations except one.

Other methods can be constructed to quantify the specificity of a gene, e.g. based on the maximal and minimal absolute values of the Z-scores. Such quantifications, however, will not scale with the number of perturbations (unless some heuristics are used), and will therefore limit the mathematical efficiency of the algorithm.

#### Estimate signaling component activity matrix

To obtain the final weight matrix determining the linear connection between the activity of an early gene and the activity of a signaling component, we multiply the activity score, the P-score, and the information score of each gene. As stated earlier, this assumes that the three scores are independent from each other, which is a restrictive assumption. To estimate the activity level of signaling component *i*, the gene expression level of each gene *k* is multiplied by the weight coefficient *Wik*, and all such terms are summed. Note, that although the scores themselves are not biased towards highly or weakly expressed genes, the multiplication process causes the results to be driven by highly expressed genes. Therefore, we divide the multiplied scores of each gene by its unperturbed activity level, giving
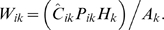



Using this notation, the activity of signaling component *i* after the perturbation of signaling component *j* is given in matrix notation by *S = WG^T^*, or specifically by
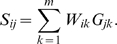



Similarly, the activity of signaling component *i* under normal conditions is given by
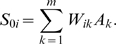



### Reverse Engineering the Network

To this point we have outlined a method to estimate the activity levels of signaling components under perturbation of each individual component, as well as under normal conditions, without measuring the signaling components themselves. Our goal is to use this knowledge to reverse engineer the interaction network between the signaling components. Any number of reverse engineering methods can be applied at this point, but we give several reasons that make a technique called modular response analysis (MRA) [19], [20] the most suitable one.

To apply MRA requires measuring the change in activity level of a representative of each component in the system, after each component is perturbed in turn. Therefore, the acquired estimated data set is a suitable input for MRA. In contrast, methods based on Bayesian learning require sufficient statistics to provide likelihood estimates, thus requiring additional experimental results [24]–[28]. Furthermore, such methods require a prior probability distribution that represents our belief or knowledge of the architecture of the network. Other approaches require integrating a large set of experimental data, either from literature or by manufacturing that data [32]–[34], and are therefore only appropriate for well studied systems.

In the context of PLACA, the change in activity of each signaling component after each perturbation is given by *R_ij_* = *S_ij_−S_0i_*. Given this matrix, MRA provides the interaction strengths between the signaling components, which is given by *r* = −[dg(*R* ^−1^)]^−1^ *R* ^−1^ [19], [20], where dg(*R*) is a diagonal matrix with a diagonal equal to that of *R*. *r_ij_* is the linear approximation of the effect component *j* has on the steady state value of component *i*. In its original context, matrix *r* holds the interaction coefficients between the signaling components, and represents the reverse engineered network. The meaning of these interaction coefficients in the context of PLACA is discussed below.

One disadvantage of MRA is that it inherently returns an interaction coefficient between every component in the network. Normally, this results in a need to set an arbitrary cutoff to determine which interactions to consider and which to ignore. A difficulty in setting such a cutoff when using PLACA arises from the fact that the activity levels are only estimated, and the contribution to the estimated activity from each gene is multiplied by an unknown constant. Thus, the significance of the actual coefficient values is uncertain apart for their sign, and a single cutoff cannot be set. This problem can be solved using data re-sampling (each time considering data from a subset of early genes), keeping only interactions that show the same sign for a significant number of the re-sampled data. Specifically, we used a jack-knifing technique, in which we ignored each gene sequentially and applied PLACA to the remaining data, obtaining a set of interaction coefficients for every pair of signaling components. For larger data-set, however, other re-sampling methods such as bootstrapping or random cross-validation may be more appropriate [42]. Using the mean and standard deviation of each coefficient and assuming a normal distribution, we keep only interactions that have a consistent sign with a 95% confidence level.

We emphasize that since PLACA relies on estimating the activity level of signaling components using downstream genes, the interactions that are deduced using PLACA do not necessarily indicate a biochemical interaction between those signaling components. Rather, inferred interactions between components indicate that they affect a shared set of genes, meaning that they share a biological function in the cell. For this reason we refer to these as *functional interactions*, where the two signaling components affect each other's function, although they may not affect each other directly. Conventional diagrams of interaction networks include nodes and arrows, where the nodes represent signaling components, and the arrows represent direct interaction between the two signaling components. To avoid confusion, we introduce a new notation, in which functional interactions are indicated by an arrow with a diamond in it.

### Neuroendocrine LβT2 Cells Experimental Methods

LβT2 cells obtained from Prof. Pamela Mellon (University of California, San Diego) were maintained at 37C/5% CO2 in humidified air in DMEM (Mediatech, Herndon, VA) supplemented with 10% fetal bovine serum (FBS) (Gemini, Calabasas, CA) and L-glutamine. Cells were grown in 10% charcoal-treated FBS (CT-FBS) (Hyclone Laboratories, Inc., Logan, UT) 18 hours before treatment with hormones or growth factor. GnRH was obtained from Bachem (Torrence, CA). The chemical inhibitors PD98059, JNK Inhibitor II (SP600125), Bisindolylmaleimide I, PP2, KN62, and AG1478 were obtained from Calbiochem. The antibodies used were anti-phospho p42/44 MAPK (Cell Signaling Technology, Beverly, MA, #9106), anti-p42/44 ERK (Cell Signaling Technology #9102).

For the western blots cells were lysed in NP-40 buffer (20mM Tris-HCl, 1%NP-40, 150mM NaCl) and protein measurements were performed with protein assay reagent (BIO RAD, Hercules, CA). 50µg of extract was separated on 10% Tris-HCl SDS-PAGE gels (BIO RAD), and transferred to PVDF membranes (Amersham, Buckinghamshire, UK). Blocking was performed for 60 minutes with 5% nonfat dry milk in Tris-buffered saline, 0.1% Tween-20 and followed by incubation with the primary antibody at 4°C overnight. Signal was visualized with goat anti-rabbit or goat anti-mouse IgG-HRP (Santa Cruz Biotechnology) using the ECL system (Amersham).

To determine the phosphorylation level of ERK with different concentrations of GnRH stimulation, pERK ELISA (Cell Signaling Technology #7177 ) and total ERK ELISA kits (Cell signaling technology #7050 ) were used according to manufacturer's instruction. LβT2 cells were stimulated with GnRH (0, 0.1, 0.3, 1, 3, 10, 100, 1000 nM) for 5 minutes, and cells were harvested. For normalization, cell lysate was divided in half; one used for pERK ELISA and another half used for total ERK ELISA. The acquired absorbance of pERK at OD450 was divided by that of total ERK providing normalized pERK activity.

Quantitative real time PCR was performed and analyzed as follows. LβT2 cells were cultured and total RNA was prepared as described in a previous study [43]. RNA was isolated using Absolutely RNA 96 well Microprep Kit (Stratagene). Approximately 2µg of the RNA was then reverse transcribed with Stratascript (Stratagene) according to manufacturer. For each reaction 1/800 of the RT reaction volume was utilized for 40 cycle three-step PCR in an ABI Prism 7900 (Applied Biosystems, Foster City, CA) in 20mM Tris pH 8.4, 50mM KCl, 5mM MgCl2, 200µM dNTPs, .5× SYBR Green I (Molecular Probes, Eugene, OR), 200nM each primer and 0.5U Platinum Taq (Invitrogen).

## Results

### Inferring a Synthetic Interaction Network

**Figure 4:**
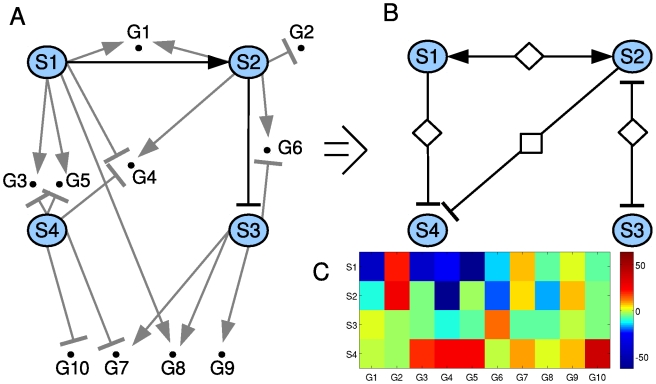
Synthetic interaction network. A. The biochemical interaction network for the synthetic network, including the four signaling components (S1–S4), and the 10 early genes they affect (G1–G10); B. The network of functional interactions between the four signaling components in the synthetic network, as inferred by PLACA. The inferred functional interactions convey the correct biochemical network; C. The heat map of the change in gene activity in all genes (X axis), as obtained from a set of simulations where each signaling component (Y axis) was perturbed. The heat map reveals which genes were involved in inferring each functional interaction.

We first tested PLACA by attempting to reverse engineer a functional network using early gene expression and perturbation experiments generated by a simulation using an arbitrary network model (Fig. 4A). The network was constructed by using well-known network motifs such as feed forward loops, bi-fans, and master regulators [3], [4], [6], and by adding a few genes that are regulated by a single signaling component. Each signaling component was assigned a characteristic activity (inhibition or activation), but exceptions were allowed and introduced to the model. A detailed description of the network model, which has four signaling components and 10 early genes, and of the simulation methods appear in the Supporting Text S1. The functional interaction network derived using PLACA is shown in Fig. 4B.

Overall, the functional reverse engineered network shows high similarity to the model that produced the early gene expression data. To explain how the various interactions were identified, consider the heat-map representing the change in gene expression following perturbations of each signaling component (Fig. 4C). First, let us consider the bi-directional positive interaction between signaling components S1 and S2. Perturbations of either S1 and S2 cause similar changes in expression in genes G1, G2, G4, and G6, which are also genes that are chosen as good estimates of both S1 and S2 expression. Genes G7, G8 and G10 also show similar behavior, further implying the bi-directional activation between S1 and S2 that is identified by PLACA. Next, genes G6, G7, G8, and G9, which are indicators of S3 activity, change in opposite ways following perturbations to S2 and S3. However, among these genes only G6 is a good indicator for S2, and thus PLACA infers that S2 inhibits S3, but not the other way around. Similarly, PLACA infers S1 inhibition of S4 through the genes G3 and G5 (which are good indicators for S4 but not for S1), and the inhibition of S4 by S2 through the genes G4 and G7.

The aim of PLACA is to reconstruct the functional interaction network from experimental data, which is often quite noisy. To test robustness to noise, we applied PLACA to the synthetic network with varying levels of noise and analyzed the similarity between the reconstructed networks. It was found that when considering only results obtained by a majority of several experiments, PLACA remains robust with noise levels of up to 20% of the mean (Supporting Fig. S1). A complete description of the methods used in this analysis is given in Supporting Text S1.

### Applying PLACA to Experimental Data

#### Reverse engineering the gonadotrope signaling network

As an example of how PLACA can be used to uncover functional interactions from experimental results, we next used PLACA in order to uncover *de-novo* part of the signaling network in the pituitary gonadotrope cell, a crucial component of the reproductive axis. Gonadotropes are endocrine cells that respond to the neuropeptide gonadotropin-releasing hormone (GnRH), by increasing the biosynthesis of luteinizing hormone (LH) and follicle-stimulating hormone (FSH). GnRH activates a G protein-coupled receptor on the surface of the gonadotrope, resulting in rapid activation of multiple kinases and signaling proteins. These kinases modulate the activity and localization of transcription factors, resulting in the induction of an early gene program. The early gene program has been investigated in considerable detail using GnRH-stimulated LβT2 gonadotropes [44], [45].

The connectivity between the kinases, transcription factors, and other signaling proteins in gonadotropes has been extensively studied but remains incompletely understood. To address this problem, we assayed the early gene activity after individual perturbation of six different kinases using chemical inhibitors (Supporting Table S1). These kinases were chosen because they are known to affect the signaling pathway, and because they are believed to only affect a single component in the pathway. In these experiments, LβT2 gonadotropes were treated with 100nM GnRH for 45 minutes in the absence or presence of individual inhibitors.

This time point represents the peak of induction for a majority of the genes assayed, and represents a time point in which early genes are highly expressed, while genes that require de-novo transcriptions (e.g. genes that are activated by the early genes) can only begin synthesizing significant amounts of mRNA. This conclusion was reached both by analysis of estimated rate constants, as well as by experimental data. Significant early gene mRNA accumulation can be detected approximately 20 minutes following stimulus (data not shown). It follows that for significant amounts of protein to be produced and affect mRNA accumulation of additional genes would require an additional 30 minutes at least. Additionally, blocking new protein synthesis, using cycloheximide, does not change the rate of accumulation at the 45 minute time point (data not shown), showing that at this early time these genes are not affected by other genes that were not measured.

**Figure 5:**
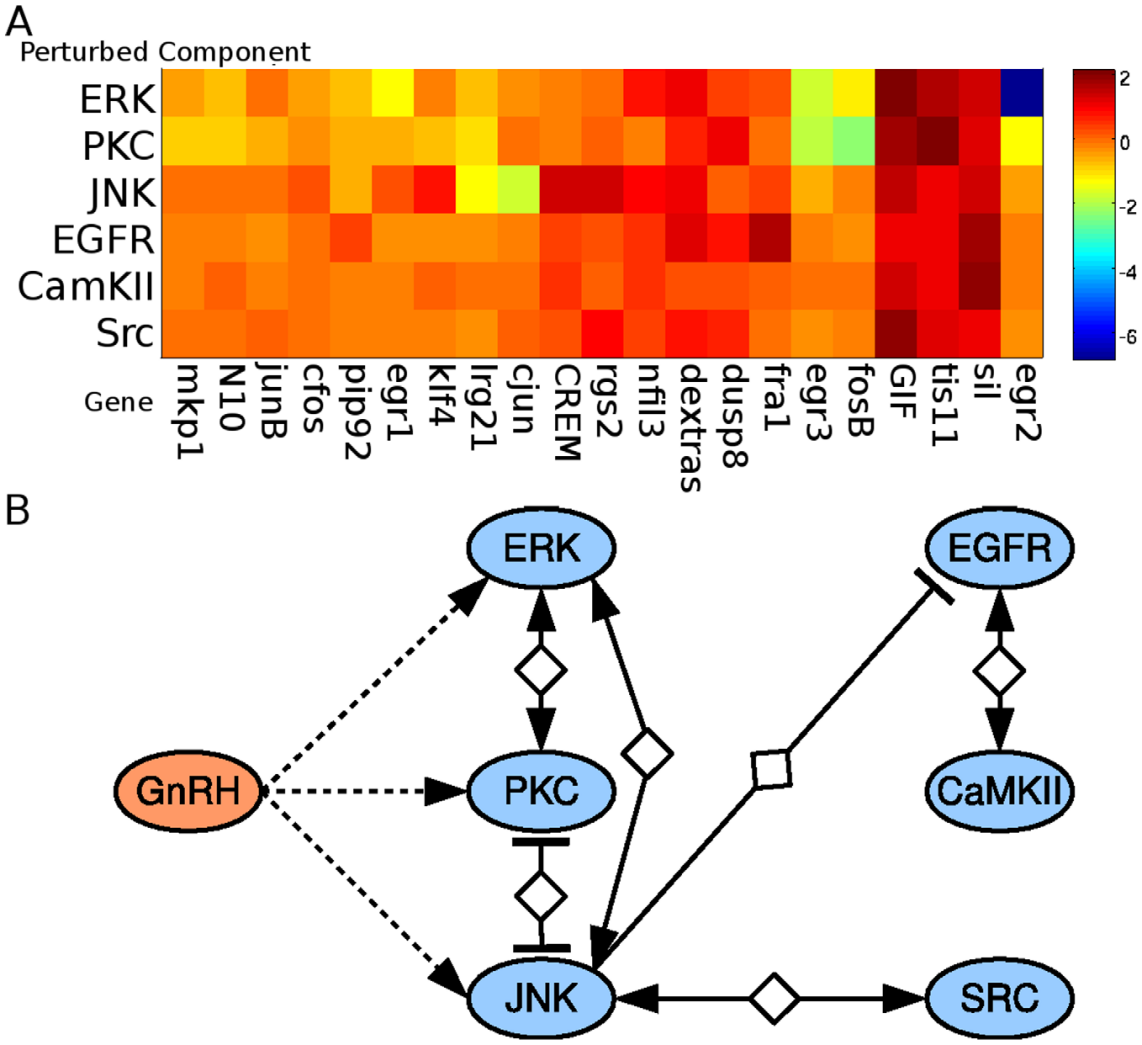
Applying PLACA to experimental results in the gonadotrope. A. The clustered heat map of the log fold change in gene activity for 21 genes (X axis), as obtained from a single experiment (experiment #6), where LβT2 gonadotropes were treated with one of six chemical inhibitors acting on signaling components (Y axis). The fold change is the gene activity in the presence of both inhibitor and GnRH divided by the gene activity with GnRH alone; B. The inferred functional network in the Gonadotrope. PLACA was applied to the experimental data from five independent experiments. The functional network represents signaling components that present a statistically significant functional interaction. The interactions between ERK and PKC, between JNK and Src, and between EGFR and CaMKII were previously seen experimentally, and we have found experimental evidence for the validity of the functional inhibition of EGFR by JNK.

We chose to assay a group of early genes based on their high response to GnRH in previous studies [44], [45]. Measurement of transcript levels were performed by SYBR green quantitative real time PCR [43]. This experiment was performed five independent times. Fig. 5A shows the clustered heat map of the measured activity in log-scale, as obtained from a single experiment, where the clustering is performed both on the genes and on the perturbations. The raw data for all five experiments is available as Supporting Dataset S1.

Analysis using PLACA produced the functional interaction network shown in Fig. 5B, in which we consider only interactions that were predicted by at least four out of the five independent experiments. This filtering is done in order to minimize the number of false interactions detected, using the post-processing procedure explained in the Supporting text S1. The resulting network has 11 interactions, out of 30 possible interactions. 8 interactions are positive and 3 are negative. 10 of the interactions are involved in bi-directional functional interactions, where the two components have a similar functional effect on each other. The probability of obtaining 11 such interactions or more in random networks is less than 0.002.

Application of PLACA to the gonadotrope cell identified the positive bi-directional interaction between Src and JNK. Studies in the similar aT3-1 cells have demonstrated that JNK is activated in a pathway downstream of Src [46]. Additionally, we performed experiments based on this reverse-engineered network that show that JNK inhibition attenuates Pyk2 activation (Supporting Fig. S2). Additionally, Src is known to activate Pyk2 [46], thus completing the bi-directional functional interaction.

PLACA also identified a strong positive bi-directional interaction between ERK and PKC, which was seen in all five experiments. It is known that PKC activation is crucial for ERK pathway activity in many G-protein coupled receptor cell systems [47], [48], and it was also shown that pharmacological inhibitors of PKC lead to partial inhibition of ERK activity in LβT2 cells [49], supporting the inferred positive interaction. We tried to ascertain if the interaction is the results of direct biochemical interaction, and found no effect of PKC inhibition on ERK activation after a 15 minutes exposure (Supporting Fig. S3). This result suggests that the functional interaction is likely due to a convergence of these two pathways. This is supported by data showing that PKC inhibition blocks the translocation of active ERK to the nucleus [49].

A positive functional interaction between CaMKII and EGFR is also predicted by PLACA. This interaction is corroborated by previous studies in other cell lines. These reports have shown that EGFR activation increases CaMKII activity through a calcium-Calmodulin dependent mechanism [50], [51].

#### Experimental confirmation of PLACA prediction

One interesting novel interaction that was identified by PLACA is the functional inhibition of EGFR by JNK. This interaction appeared in four experiments, and was significant in three. This putative functional interaction allows testing the limits of PLACA's predictive power since we consider this prediction *not* to be highly robust.

A convenient method to identify the genes that contribute most to a specific interaction is to consider the individual contribution of each gene to the activity estimate of the affected signaling component given by 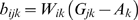, where 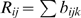. For this interaction, we looked for genes that contributed a positive term to the estimate of EGFR (*j*) activity after the negative perturbation of JNK (*i*) in every experiment. The gene that contributed the largest term in all experiments was *klf4*. In the original experiments, it was seen that *klf4* is indeed hyper-induced by GnRH after inhibition of JNK, and is attenuated following inhibition of EGFR (see Fig. 5A). PLACA predicted an inhibitory functional interaction, where JNK inhibits EGFR.

**Figure 6:**
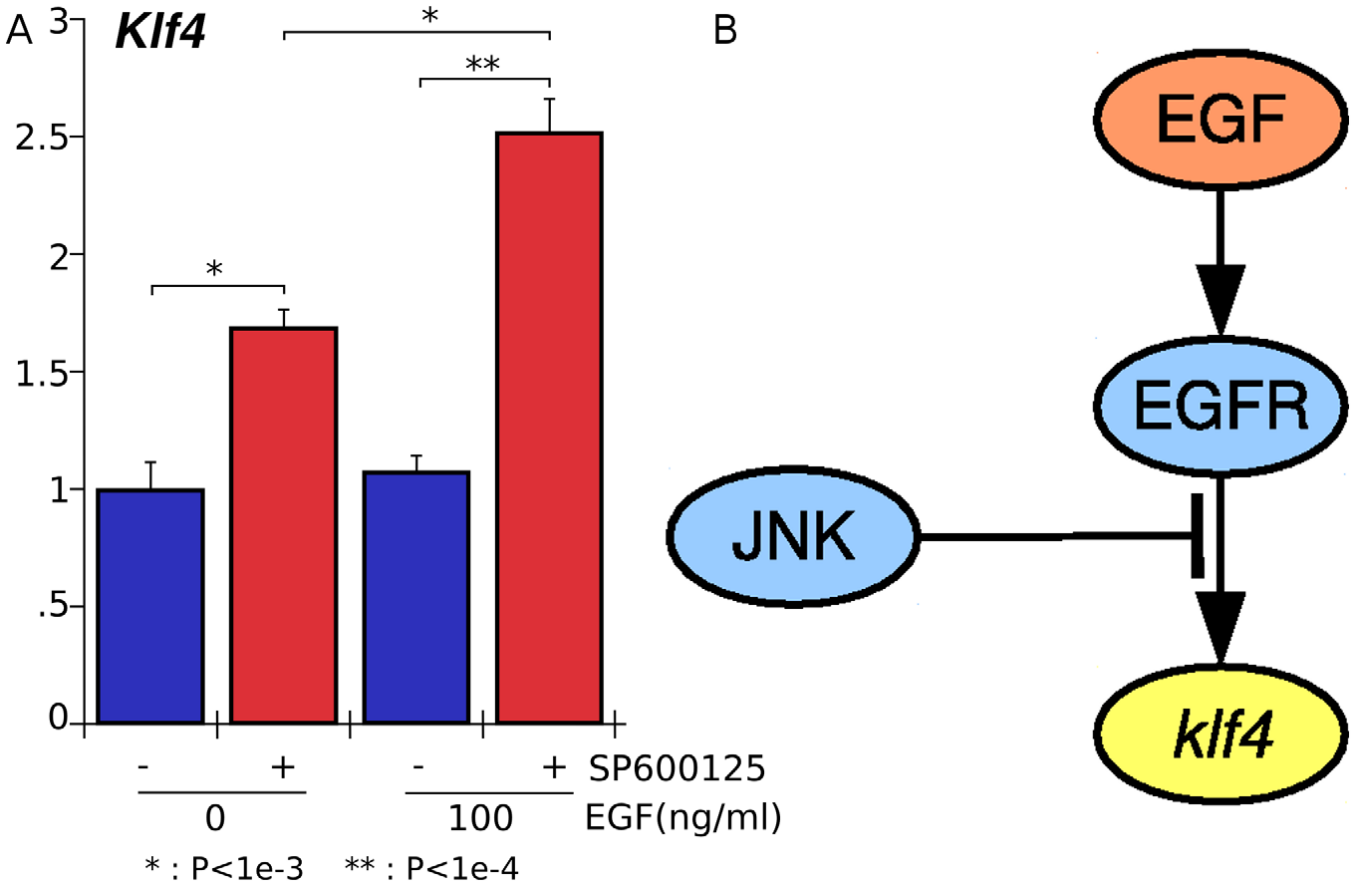
JNK represses EGF-Stimulation of *klf4*. A. Experimental results showing that JNK repression enhances EGF-stimulation of *klf4*. LβT2 cells were pre-incubated with SP600125 (JNK Inhibitor) for 30 minutes and treated with different concentration of EGF for 45 minutes. The levels of *klf4* were measured by quantitative PCR and rps11 was used for normalization. EGF alone causes a slight induction of *klf4*, and inhibition of JNK results in a more significant induction. With concurrent JNK inhibition and EGF stimulation, a synergistic effect can be seen; B. The proposed biochemical interaction network involving JNK, EGF, EGFR, and *klf4* that mediates the functional repression of EGFR by JNK.

We proceeded to experimentally test this interaction when the EGFR is directly activated by its ligand (Fig. 5B, Fig. 6B). Notably, this is a non-trivial prediction test, since the PLACA analysis was based on GnRH activation of its receptor and inhibition of EGFR and JNK *separately*. The prediction that JNK suppresses the functional effects of EGRF is now tested by using an activator of EGFR and a JNK inhibitor jointly. The network predicts that inhibition of JNK will augment the functional response to EGFR activation, with that response being activation of *klf4*. Fig. 6 shows the levels of *klf4*, as measured by quantitative PCR, after treatment of LβT2 cells with a JNK inhibitor and varying levels of EGF, alone and together. These data indicate that JNK inhibits the effects of EGF receptor signaling, a result that is consonant with the predictions made by PLACA.

## Discussion

In this manuscript we introduced an algorithm that uses changes in the level of early gene induction in order to estimate the activity of unmeasured upstream signaling components, and then infer the functional interactions between the signaling components. The algorithm is useful for translating recent advances in technology that utilize high throughput measurement of gene activity into novel insights of cellular network design and signal processing. Despite the introduction of methods that allow to obtain high throughput data of the levels of protein activity state (multiplexed ELISA, DNA binding assays), in some cases such measurements may be impractical as in the case of scaffold proteins, some transcription factors, and kinases with an unknown number of active states. As gene expression assays and RNAi component perturbations are both sequence dependent, they are readily performed for any target and PLACA is suitable for systematic large scale reverse engineering of any signaling network. The experiments suggested by PLACA are easy to design and feasible to perform.

In addition to the problem of measuring the signaling components themselves, in some biological setups, such as the one discussed here, measuring steady state values is not applicable, since the system becomes desensitized when exposed to a prolonged stimulus. Conventional reverse engineering techniques that rely on steady state analysis will not be able to reverse engineer such systems. Early gene analysis can help solve this problem, and PLACA can be applied in these cases to reverse engineer the network.

PLACA provides a mathematically efficient algorithm that scales linearly with the number of genes and polynomially (O(*n* ^3^)) with the number of signaling components. Large gene expression data-set, however, tend to display data-degeneracy, where multiple genes behave similarly under various experimental conditions. This problem is likely to become worse when the analysis is limited to early gene expression. However, as long as the number of sets of similarly behaving genes is larger than the number of perturbed signaling components PLACA will treat the genes in each set as one, and thus still be applicable.

It should be noted that like many reverse engineering methods, the output of PLACA is the network of *functional interactions* between the signaling components, and not direct biochemical interactions. Such interactions, however, indicate that both signaling components affect a mutual set of genes, and thus provide a useful level of abstraction that gives an indication to which pathways interact in a non-trivial way. Further experiments are needed in order to identify the molecular mechanisms underlying the functional interactions. Still, PLACA provides a list of the genes that are most likely involved in each interaction, further reducing the ambiguity in the meaning of the functional interaction, and suggesting a means to perform follow-up experiments to validate the interaction.

A potential problem may arise from using gene activity levels as linear estimates. The activity of some early genes was shown to follow a linear response curve for a large range of signaling activity [36] (see also Fig. 1), and the assumption that many pathways work within the linear range of stimulus response is a classical pharmacological concept. On the other hand, in cases where linearity was not observed, this assumption is only valid when the changes in activity levels are small. However, experiments are normally designed to produce statistically significant results, and the changes in activity levels are therefore large. This is a problem that arises in most reverse engineering method relying on perturbations, and may skew the results.

Another disadvantage of the proposed algorithm involves the inability to compare the inferred network to other possible networks, in a similar way to statistical learning algorithms. However, as mentioned in the methods section, after obtaining the estimated activity of the signaling components it is possible to apply a different reverse engineering algorithm such as a Bayesian learning algorithm. Such a methodology will require further experiments, but will also reveal more information about the regulatory network.

The experimental results shown here were shown as an example of the ease of use of PLACA, and its applicability to experimental data. PLACA uncovered much of the known interaction network of the subsystem that was tested, and uncovered several novel interactions. These interactions must be further explored in order to understand the biochemical interactions underlying them, and in order to understand their biological significance.

PLACA offers a method to exploit the growing amounts of data that are produced by high-throughput experiments. At the same time PLACA also offers a new level of abstraction that is manifested by functional interactions. This level of abstraction can be extremely useful in the experimental, pharmacological, and theoretical levels. It can extend our understanding of emergent phenomena in regulatory networks, and offer new insights into the effects of drugs, hormones and pathogens on cells.

## Supporting Information

Dataset S1 Gene expression results of GnRH-induced early genes in LβT2 cells. Five experiments were performed, measuring 18 to 24 early genes. The data set contains the gene expression values with GnRH treatment, and with a combination treratment of GnRH and an additional chemical inhibitior, as well as the standard deviation of the measurements.
(0.11 MB XLS)

Figure S1 Network similarity score vs. signal to noise ratio. A. Functional networks were inferred either from a single simulation of the synthetic network (circles) or by at least three out of five simulations (squares). The similarity scores were computed using the interaction coefficients values (open symbols), or using the sign of the interaction coefficients (full symbols). Using multiple experiments, PLACA is robust up to signal to noise ratios (SNRs) of 5; B–E. The inferred functional interaction network obtained from majority rule (three out of five experiments) with varying values of SNRs. As noise levels increase, fewer interactions are identified, but erroneous interactions are seldom introduced.
(0.59 MB TIF)

Figure S2 Potential Biochemical Interaction between the Src and JNK Pathways. LβT2 cells were either pretreated with 50µM SP600125 (JNK inhibitor) for 30 minutes or left untreated. They were then treated with 100nM GnRH for 0, 15, or 30 minutes. A. Pyk2 tyrosine phosphorylation was measured by western blotting with an anti-phosphotyrosine antibody. B. Tyrosine phosphorylation was quantified as fold change relative to vehicle treated samples for cells treated with GnRH alone (solid line), and for cells treated with SP600125 and GnRH (dotted line). Pyk2 is a known substrate of Src. Inhibition of JNK attenuates the Pyk2 response to GnRH, suggesting that JNK activates this response and is functionally linked to Src, as identified by PLACA.
(0.62 MB TIF)

Figure S3 GnRH Activates the ERK and PKC Pathways in Parallel. LβT2 cells were incubated with either the chemical inhibitor PD98059 (ERK inhibitor, 50µM), SP600125 (JNK inhibitor, 50µM), or BIM I (PKC inhibitor, 10µM) for 30 minutes. Cells were then treated with 100nM GnRH for 15 minutes and lysed. The activation of ERK, PKC, and JNK was measured by Western Blot using phospho-ERK, phospho-JNK, and phospho-PKD antibodies. The phospho-PKD site is a direct PKC phosphorylation substrate site. Each chemical inhibitor inhibits one kinase, suggesting that there is no direct interaction between the kinases. This experiment was performed three times with similar results. The asterisk signifies a non-specific band.
(1.07 MB TIF)

Table S1 The chemical inhibitors used in the experiments and the kinases they inhibit
(0.02 MB XLS)

Text S1 Detailed description and methods. Describes the post-processing stage of the algorithm, the synthetic model equations and methods, the parameters used in the simulations, and the details of the noise analysis methods.
(0.06 MB PDF)
