# Supplementary figures and images for: Plato's Cave Algorithm: Inferring Functional Signaling Networks from Early Gene Expression Shadows
Source: PLoS Comput Biol. 2010 Jun 24;6(6):e1000828. doi: 10.1371/journal.pcbi.1000828 (PMC2891706; doi:10.1371/journal.pcbi.1000828)

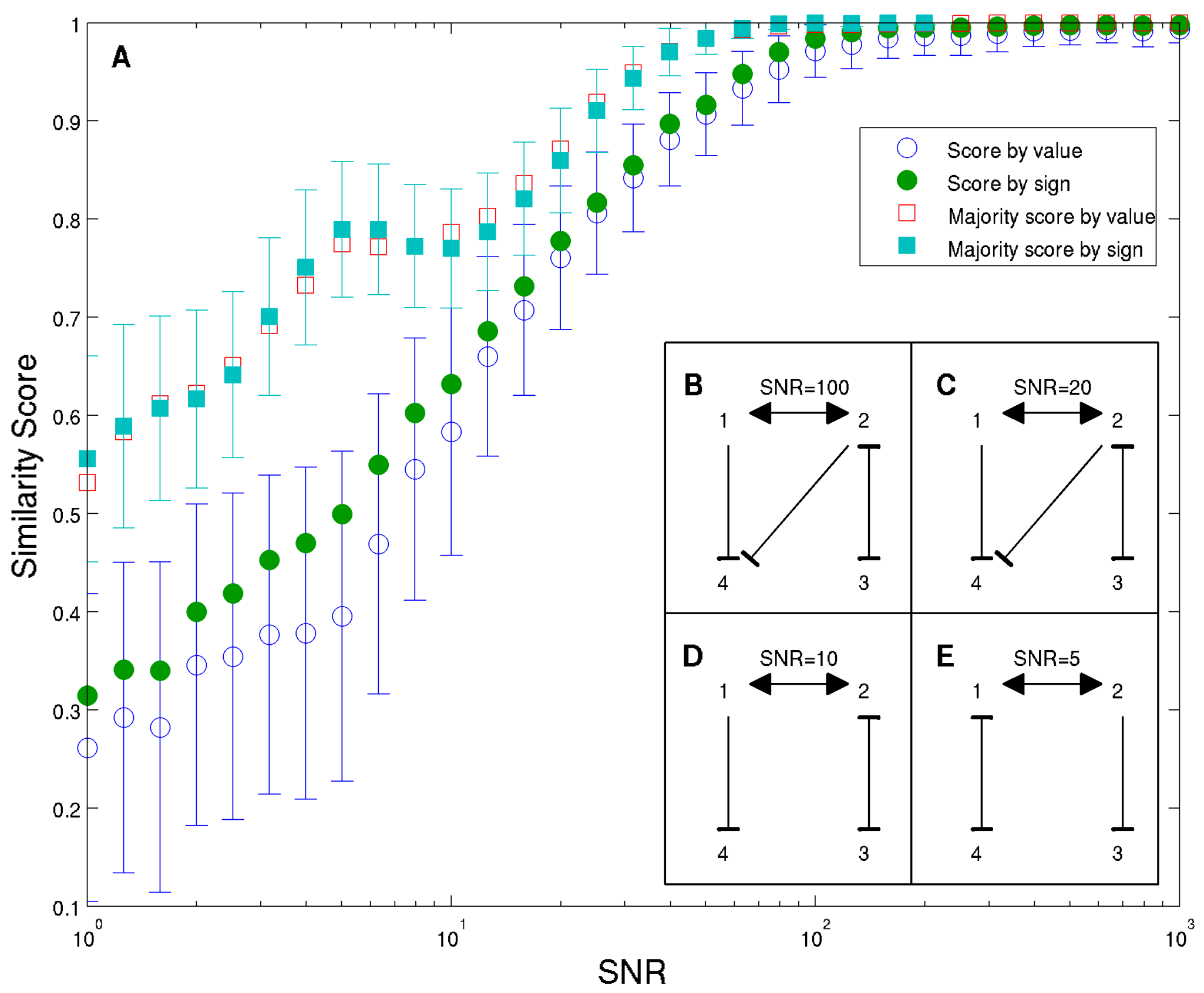

Supplement: Figure S1 — Network similarity score vs. signal to noise ratio. A. Functional networks were inferred either from a single simulation of the synthetic network (circles) or by at least three out of five simulations (squares). The similarity scores were computed using the interaction coefficients values (open symbols), or using the sign of the interaction coefficients (full symbols). Using multiple experiments, PLACA is robust up to signal to noise ratios (SNRs) of 5; B–E. The inferred functional interaction network obtained from majority rule (three out of five experiments) with varying values of SNRs. As noise levels increase, fewer interactions are identified, but erroneous interactions are seldom introduced. (0.59 MB TIF) [file pcbi.1000828.s002.tif]

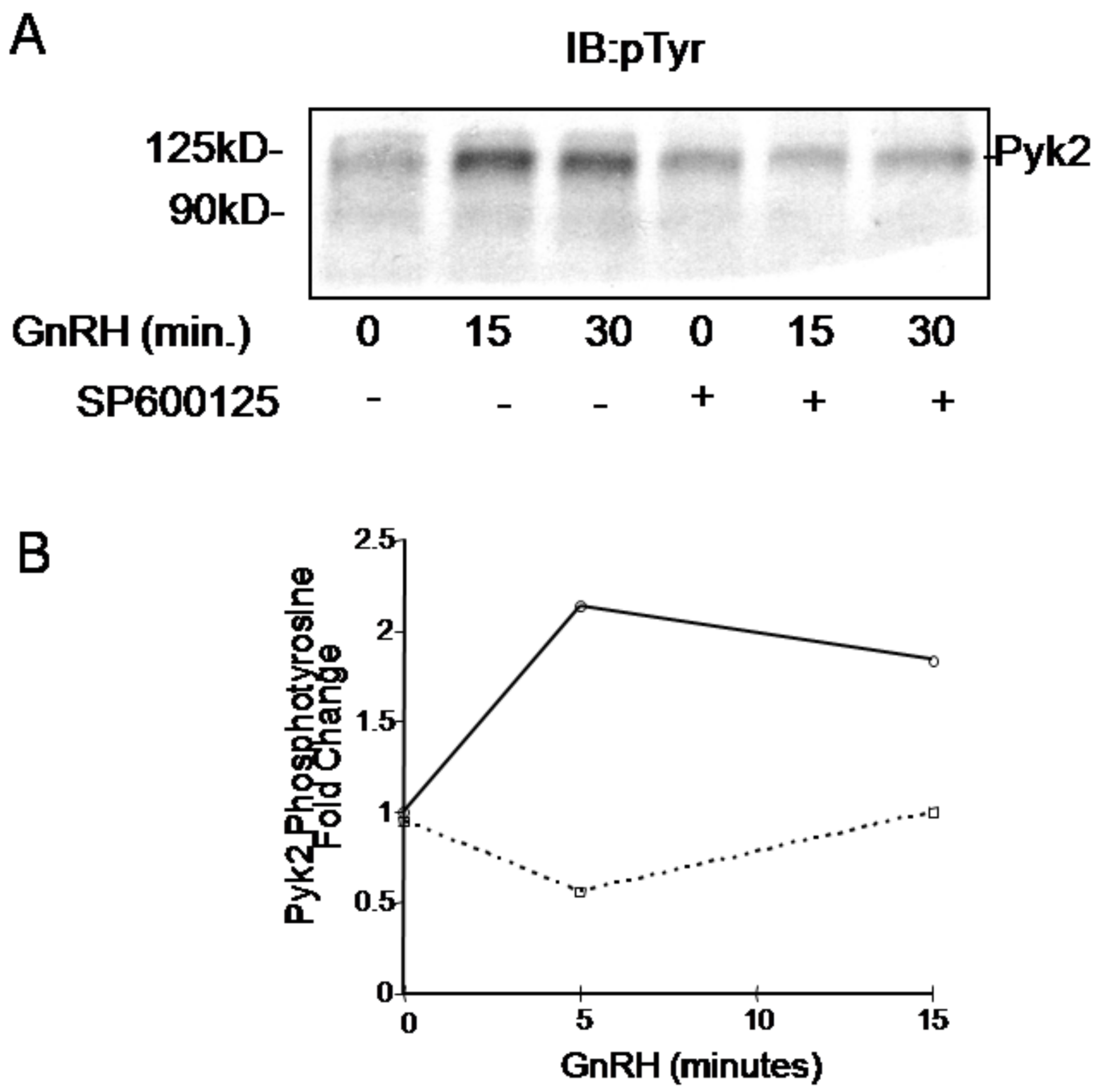

Supplement: Figure S2 — Potential Biochemical Interaction between the Src and JNK Pathways. LβT2 cells were either pretreated with 50µM SP600125 (JNK inhibitor) for 30 minutes or left untreated. They were then treated with 100nM GnRH for 0, 15, or 30 minutes. A. Pyk2 tyrosine phosphorylation was measured by western blotting with an anti-phosphotyrosine antibody. B. Tyrosine phosphorylation was quantified as fold change relative to vehicle treated samples for cells treated with GnRH alone (solid line), and for cells treated with SP600125 and GnRH (dotted line). Pyk2 is a known substrate of Src. Inhibition of JNK attenuates the Pyk2 response to GnRH, suggesting that JNK activates this response and is functionally linked to Src, as identified by PLACA. (0.62 MB TIF) [file pcbi.1000828.s003.tif]

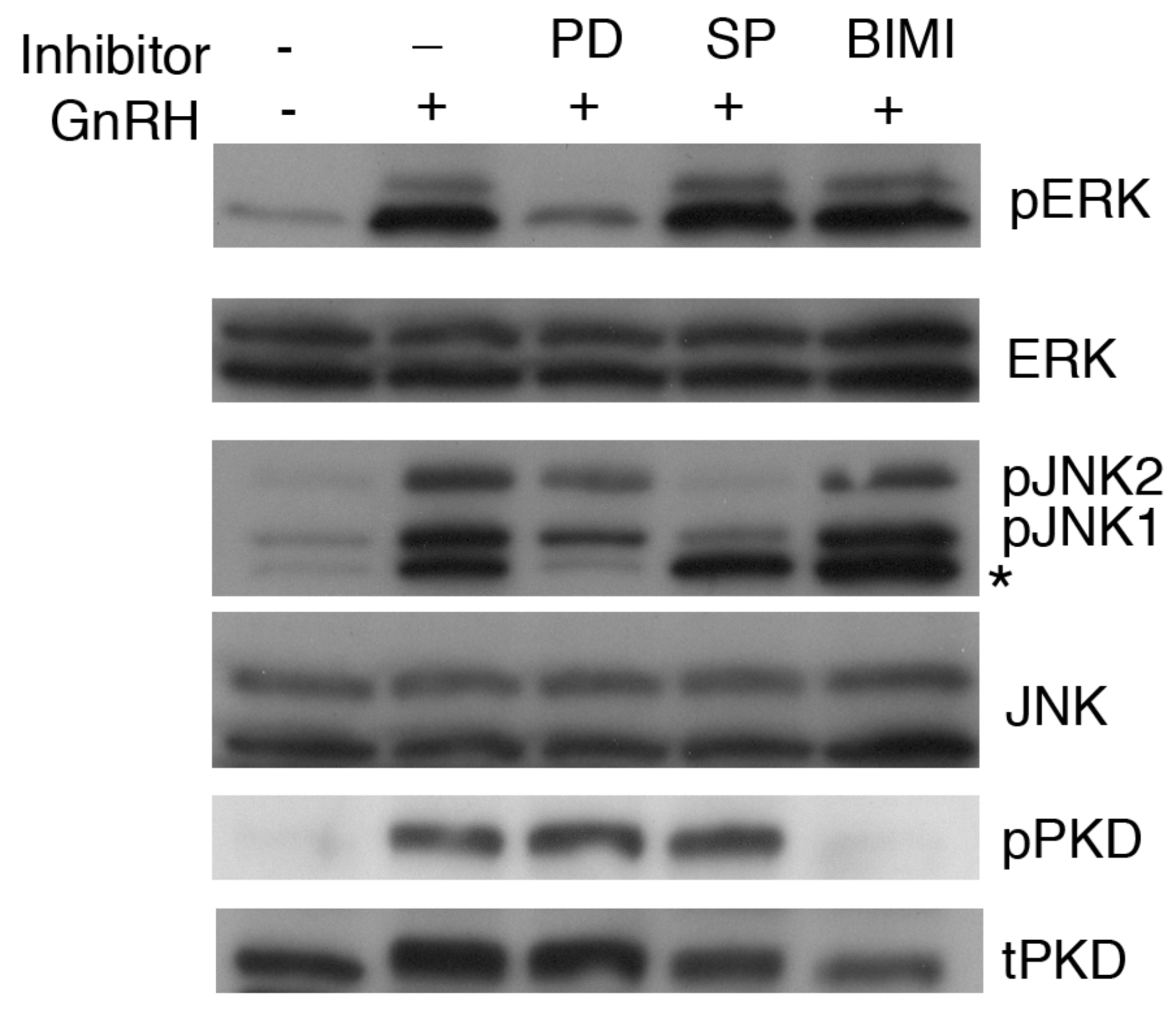

Supplement: Figure S3 — GnRH Activates the ERK and PKC Pathways in Parallel. LβT2 cells were incubated with either the chemical inhibitor PD98059 (ERK inhibitor, 50µM), SP600125 (JNK inhibitor, 50µM), or BIM I (PKC inhibitor, 10µM) for 30 minutes. Cells were then treated with 100nM GnRH for 15 minutes and lysed. The activation of ERK, PKC, and JNK was measured by Western Blot using phospho-ERK, phospho-JNK, and phospho-PKD antibodies. The phospho-PKD site is a direct PKC phosphorylation substrate site. Each chemical inhibitor inhibits one kinase, suggesting that there is no direct interaction between the kinases. This experiment was performed three times with similar results. The asterisk signifies a non-specific band. (1.07 MB TIF) [file pcbi.1000828.s004.tif]
