# Supplementary material for: Plato's Cave Algorithm: Inferring Functional Signaling Networks from Early Gene Expression Shadows
Source: PLoS Comput Biol. 2010 Jun 24;6(6):e1000828. doi: 10.1371/journal.pcbi.1000828 (PMC2891706; doi:10.1371/journal.pcbi.1000828)
Supplement: Text S1 — Detailed description and methods. Describes the post-processing stage of the algorithm, the synthetic model equations and methods, the parameters used in the simulations, and the details of the noise analysis methods. (0.06 MB PDF) [file pcbi.1000828.s006.pdf]

# Supplementary text for the paper: Plato's Cave Algorithm: Inferring Functional Signaling Networks from Early Gene Expression Shadows

Yishai Shimoni*, Marc Fink*,Soon-gang Choi, and Stuart Sealfon+
Department of Neurology and Center for Translational Systems Biology,
Mount Sinai School of Medicine, New York, NY
* These authors contributed equally to this work
+ Email: stuart.sealfon@mssm.edu

## I. POST-PROCESSING STAGE DETAILED METHODS

When multiple experiments are performed using similar perturbations of the same signaling components, the results from these experiments can be used to further refine the interaction network, by choosing only interactions which were inferred from a large majority (or all) of the experiments, and disregarding the rest. Given enough experiments, this method ensures that only correct interactions are chosen, and random ones caused by over-fitting noise are removed. To determine how many experiments should be considered we must determine the statistical significance of the results obtained by such a cutoff. Given $L$ experiments from which we derive $L$ networks, we create many ($10^6$) sets of $L$ random networks, each set containing the same number of positive and negative interactions as the networks derived from the experimental data. For each set we count how many interactions have the same sign in $C$ out of the $L$ experiments ($C \leq L$). We thus create the probability distribution of obataining at least $n$ agreeing interactions in at least $C$ of the experiments. Finally, we examine the number of agreeing interactions for every value of $C$ in the networks derived from the experimental data, and choose the value that gives the highest statistical significance, namely the value that is most unlikely randomly given that value of $C$. In the experimental results used in this paper this analysis resulted in using 4 out of the 5 experiments, and obtaining 11 interactions. The probability that 5 similar random networks (with the same number of positive and negative interactions as the experimental data) will results in 11 interactions in at least 4 out of the 5 networks is less than 0.002.

## II. SYNTHETIC MODEL DESCRIPTION AND METHODS

We constructed a synthetic model that consists of four signaling components and ten early genes. The signaling components are simulated as proteins that have active and inactive forms, and can switch between them. A schematic representation of this regulatory network is presented in Fig. 3A in the main text, in which signaling components are denoted by $S_j$ ($j = 1..4$), and early genes by $G_n$ ($n = 1..10$). The steady state values of this network were obtained both for the unperturbed network and for the network when one of the signaling components is perturbed, using an ode solver in Matlab 7.7.0.471 (R2008b). Perturbation were performed by changing either the activation rate or inactivation rate of the signaling components by 5-10%, which simulates adding a kinase inhibitor or a protease inhibitor, respectively. To these steady state values we added Gaussian noise with a mean of zero and a standard deviation which is some percentage of the mean, which defines the signal to noise ratio (SNR).

Below we introduce the ordinary differential equations used to simulate the synthetic network. We denote the concentration of the mRNA of gene $n$ by $m_n$ ($n = 1..10$), the active and inactive forms of signaling component $j$ by $x_{ja}$ and $x_{ji}$, respectively ($j = 1..4$). The rate constant for activation and inactivation of signaling component $j$ are $a_{xj}$ and $i_{xj}$. The rates at which molecules are generated (translated or transcribed) and degraded are denoted by $g$ or $d$, respectively, with the molecule name as subscript. The activation and repression of the genes by the signaling components is done using a Michaelis-Menten like function, where we assume a single promoter site that can be bound by a single transcription factor. The rate constant for the activation and repression of gene $n$ by signaling component $j$ is given by $k_{axjgn}$ or $k_{rxjgn}$, respectively. Finally, the ratio between the basal and maximal gene production for gene $n$ is denoted by $r_n$

$$\frac{d(x_{1i})}{dt} = g_{x1} - a_{x1}x_{1i} + i_{x1}x_{1a} - d_{x1i}x_{1i} \tag{S1}$$

$$\frac{d(x_{1a})}{dt} = a_{x1}x_{1i} - i_{x1}x_{1a} - d_{x1a}x_{1a} \tag{S2}$$

$$\frac{d(x_{2i})}{dt} = g_{x2} - a_{x2}x_{2i}x_{1a} + i_{x2}x_{2a} - d_{x2i}x_{2i} \tag{S3}$$

$$\frac{d(x_{2a})}{dt} = a_{x2}x_{2i}x_{1a} - i_{x2}x_{2a} - d_{x2a}x_{2a} \tag{S4}$$

$$\frac{d(x_{3i})}{dt} = g_{x3} - a_{x3}x_{3i} + i_{x3}x_{3a}x_{2a} - d_{x3i}x_{3i} \tag{S5}$$

$$\frac{d(x_{3a})}{dt} = a_{x3}x_{3i} - i_{x3}x_{3a}x_{2a} - d_{x3a}x_{3a} \tag{S6}$$

$$\frac{d(x_{4i})}{dt} = g_{x4} - a_{x4}x_{4i} + i_{x4}x_{4a} - d_{x4i}x_{4i} \tag{S7}$$

$$\frac{d(x_{4a})}{dt} = a_{x4}x_{4i} - i_{x4}x_{4a} - d_{x4a}x_{4a} \tag{S8}$$

$$\frac{d(m_1)}{dt} = g_{m1}\frac{k_{ax1g1}x_{1a} + k_{ax2g1}x_{2a} + r_1}{k_{ax1g1}x_{1a} + k_{ax2g1}x_{2a} + 1} - d_{m1}m_1 \tag{S9}$$

$$\frac{d(m_2)}{dt} = g_{m2}\frac{1}{k_{rx2g2}x_{2a} + 1} - d_{m2}m_2 \tag{S10}$$

$$\frac{d(m_3)}{dt} = g_{m3}\frac{k_{ax1g3}x_{1a} + r_3}{k_{ax1g3}x_{1a} + k_{rx4g3}x_{4a} + 1} - d_{m3}m_3 \tag{S11}$$

$$\frac{d(m_4)}{dt} = g_{m4}\frac{k_{ax2g4}x_{2a} + r_4}{k_{ax2g4}x_{2a} + k_{rx1g4}x_{1a} + k_{rx4g4}x_{4a} + 1} - d_{m4}m_4 \tag{S12}$$

$$\frac{d(m_5)}{dt} = g_{m5}\frac{k_{ax1g5}x_{1a} + r_5}{k_{ax1g5}x_{1a} + k_{rx4g5}x_{4a} + 1} - d_{m5}m_5 \tag{S13}$$

$$\frac{d(m_6)}{dt} = g_{m6}\frac{k_{ax2g6}x_{2a} + r_6}{k_{ax2g6}x_{2a} + k_{rx3g6}x_{3a} + 1} - d_{m6}m_6 \tag{S14}$$

$$\frac{d(m_7)}{dt} = g_{m7}\frac{k_{ax3g7}x_{3a} + r_7}{k_{ax3g7}x_{3a} + k_{rx4g7}x_{4a} + 1} - d_{m7}m_7 \tag{S15}$$

$$\frac{d(m_8)}{dt} = g_{m8}\frac{k_{ax1g8}x_{1a} + k_{ax3g8}x_{3a} + r_8}{k_{ax1g8}x_{1a} + k_{ax3g8}x_{3a} + 1} - d_{m8}m_8 \tag{S16}$$

$$\frac{d(m_9)}{dt} = g_{m9}\frac{k_{ax3g9}x_{3a} + r_9}{k_{ax3g9}x_{3a} + 1} - d_{m9}m_9 \tag{S17}$$

$$\frac{d(m_{10})}{dt} = g_{m10}\frac{1}{k_{rx4g10}x_{4a} + 1} - d_{m10}m_{10} \tag{S18}$$

For completeness, the rate contants values that were used for the unperturbed simulation follow.
Activation rate constants: $a_{x1} = 0.01$, $a_{x2} = 0.01$, $a_{x3} = 0.2$, $a_{x4} = 0.2$.
Inactivation rate constants: $i_{x1} = 0.01$, $i_{x2} = 0.1$, $i_{x3} = 0.1$, $i_{x4} = 0.1$.
Degradation rate constants: $d_{x1i} = 0.005$, $d_{x2i} = 0.005$, $d_{x3i} = 0.013$, $d_{x4i} = 0.015$, $d_{x1a} = 0.005$, $d_{x2a} = 0.005$, $d_{x3a} = 0.013$, $d_{x4a} = 0.015$, $d_{m1} = 0.005$, $d_{m2} = 0.0067$, $d_{m3} = 0.004$, $d_{m4} = 0.002$, $d_{m5} = 0.003$, $d_{m6} = 0.0025$, $d_{m7} = 0.0075$, $d_{m8} = 0.004$, $d_{m9} = 0.008$, $d_{m10} = 0.00333333$.
Translation rate constants: $g_{x1} = 0.5$, $g_{x2} = 0.5$, $g_{x3} = 1$, $g_{x4} = 2$.
Transcription rate constants: $g_{m1} = 0.8$, $g_{m2} = 0.85$, $g_{m3} = 0.65$, $g_{m4} = 0.9$, $g_{m5} = 0.7$, $g_{m6} = 0.6$, $g_{m7} = 0.8$, $g_{m8} = 0.95$, $g_{m9} = 0.7$, $g_{m10} = 0.65$.
Michaelis-Menten constants for strength of ativation and repression: $k_{ax1g1} = 0.025$, $k_{ax2g1} = 0.0125$, $k_{rx2g2} = 0.0125$, $k_{ax1g3} = 0.025$, $k_{rx4g3} = 0.011$, $k_{ax2g4} = 0.0125$, $k_{rx1g4} = 0.025$, $k_{rx4g4} = 0.0111$, $k_{ax1g5} = 0.025$, $k_{rx4g5} = 0.01111$, $k_{ax2g6} = 0.0125$, $k_{rx3g6} = 0.06667$, $k_{ax3g7} = 0.0666667$, $k_{rx4g7} = 0.0111111$, $k_{ax1g8} = 0.025$, $k_{ax3g8} = 0.0666667$, $k_{ax3g9} = 0.066667$, $k_{rx4g10} = 0.0111111$.
Basal gene activity ratios: $r_1 = 0.1$, $r_3 = 0.1$, $r_4 = 0.1$, $r_5 = 0.1$, $r_6 = 0.1$, $r_7 = 0.1$, $r_8 = 0.1$, $r_9 = 0.2$. Note that the basal gene activity levels are only needed for activated genes, and therefore $r_2$ and $r_{10}$ are not defined.

## III. NOISE ANALYSIS METHODS

We applied PLACA to the synthetic network with various levels of signal to noise ratios (SNRs), in order to examine the differences between the inferred networks at different levels of noise. To quantify the similarity between two networks that are inferred by PLACA, we denote the vector holding the interaction coefficients of one network by $X$ (holding $n(n-1)$ interaction coefficients, where $n$ is the number of components in the network), and the second network by $Y$. Assuming that each coefficient is independent, the similarity between the two networks is given by the normalized Euclidean inner product of the two vectors, which is defined as

$$\langle X \cdot Y \rangle = \frac{\sum_{i=1}^{n^2} X_i Y_i}{\sqrt{\sum_{i=1}^{n^2} X_i^2}\sqrt{\sum_{i=1}^{n^2} Y_i^2}}. \quad \text{(S19)}$$

Supporting Fig. S1A shows the mean similarity score as a function of the SNR, when applied to the coefficient values (denoted score by value, open circles), and when only the sign of the coefficients were considered (denoted score by sign, full circles). The results were obtained from $10^3$ experiments, and similarity scores were evaluated between each pair, giving approximately $5 \cdot 10^5$ scores for each SNR value. The mean score is above 0.8 for SNR values larger than 20, and falls rapidly as the noise levels increase. Supporting Fig. S1A also shows the score for networks obtained after simulating five experiments and using majority rule filtering, when the scoring is done by value (squares) and by sign (full squares). It can be seen that using multiple experiments makes the method extremely robust to noise, with a similarity score of more than 0.8 even for SNR levels approaching 5.

Supporting Fig. S1B-E are the functional interaction networks reconstructed by PLACA after simulating five experiments, and leaving only interaction inferred by 3/5 of the experiments. The networks are derived from simulations with SNR values of 100, 20, 10, and 5, respectively, and show that PLACA correctly infers the functional interaction of the toy network for low noise levels. As the noise levels increase, interactions are removed, but erroneous interactions are seldom introduced. Even at low SNR values, PLACA inferrs a functional network that shows high semblance to the original biochemical network.

[1] T. Yuen, W. Zhang, B. J. Ebersole, and S. C. Sealfon, G Protein Pathways: Pt C, Effector Mechanisms **345**, 556 (2002).
